# Drop foot post-ECMO, subsequently complicated by third-degree burns: A case report based on user portrait and health management journey map

**DOI:** 10.1097/MD.0000000000044008

**Published:** 2025-08-22

**Authors:** You Yuan, Jingwen Zhang, Yanyan Mou, Yan Zhou, Chaojin Yang, Li Li, Huiming Gao, De Su, Feiyu Yan, Rujun Hu

**Affiliations:** a Department of Critical Care Medicine, Affiliated Hospital of Zunyi Medical University, Zunyi, Guizhou Province, P.R. China; b School of Nursing, Zunyi Medical University, Zunyi, Guizhou Province, P.R. China; c Department of Nursing, Affiliated Hospital of Zunyi Medical University, Zunyi, Guizhou Province, P.R. China.

**Keywords:** ECMO complications, health management journey map, lower extremity drop foot, patient user portrait, post-ICU syndrome, rehabilitation, third-degree burn

## Abstract

**Rationale:**

The long-term complications of extracorporeal membrane oxygenation (ECMO) have not been well documented, especially the rare lower extremity drop foot (LEDF). Understanding the mechanisms and management of such complications is critical to improving patient outcomes. What is the role of patient-based on user portrait and health management journey map (HMJM) for rehabilitation management of patients with post-intensive care syndrome (PICS)?

**Patient concerns:**

We reported a case of a patient who developed LEDF after receiving ECMO for severe heart failure. The patient’s user profile revealed a 17-year-old female athlete with a past history of hypertension. After treatment with ECMO, the patient developed LEDF with loss of sensation and motor deficits resulting in third-degree burns, which were inadvertently caused by using an electric stove for heating in the winter. During her hospitalization, she experienced several medical interventions and became more sensitive to pain and dysfunction perception. After discharge from the ICU, the patient reported significant difficulties in mobility, quality of life, and mental health.

**Diagnoses:**

The diagnosis of LEDF was confirmed by clinical electromyography, third-degree burns were assessed using the burn assessment criteria, and the scale confirmed PICS.

**Interventions:**

User portrait and HMJM provided patients with personalized integrated rehabilitation care from a multidisciplinary team. This included physical and pharmacological treatment for foot drop. A skin graft was applied to the burned area. In addition, psychotherapy was received during the peri-rehabilitation period.

**Outcomes:**

Despite comprehensive interventions, the patient showed only partial recovery of foot function and required long-term rehabilitation and assistive devices for daily activities. However, mental health performance was better than before.

**Lessons:**

This case highlighted the importance of monitoring patients with ECMO for neuromuscular injuries, such as LEDF. The need for early intervention to prevent secondary injuries, such as burns. It also demonstrated the value of user portrait and HMJM in guiding individualized rehabilitation care plans for PICS.

## 1. Introduction

Extracorporeal membrane oxygenation (ECMO) is an advanced life-support technology used in intensive care unit (ICU), mainly for patients with cardiopulmonary failure. Vital signs and organ function are maintained by drawing the patient’s blood out of the body, oxygenating it and then infusing it back into the body.^[1]^ Veno-venous extracorporeal membrane oxygenation is mainly used to support respiratory function, while veno-arterial ECMO (V-A ECMO) is used to support circulatory function.^[2]^ There are some challenges and risks associated with the use of ECMO, such as bleeding, embolism, infection, renal insufficiency, lower limb ischemia, neurological damage and other complications.^[3]^ Among them, long-term unilateral limb foot drop with sensory loss and motor deficits is a relatively rare but important problem. These patients are exposed to a variety of risks in their daily lives, and these risks are mainly related to falls, skin injuries and infections.

ECMO patients treated in the ICU may develop post-intensive care syndrome (PICS), which is a long-term physical, cognitive, and psychosocial dysfunction that severely impacts a patient’s quality of life. Studies have shown that patients treated with ECMO have higher rate of newly diagnosed mental health problems after discharge than other ICU patients, with depression, anxiety and post-traumatic stress disorder being the most common mental health problems.^[4,5]^

What is the role of patient-based on user portrait and health management journey map for rehabilitation management of patients with PICS? To better understand the journey of patients with PICS after ECMO, this case used a patient user portrait and journey map to help identify the patient’s key points and needs during the treatment process. This patient developed lower extremity drop foot after receiving V-A ECMO for severe heart failure. Due to foot drop with loss of sensation, she accidentally suffered third-degree burns while using an electric stove to keep warm in the winter. During her hospitalization, she underwent several medical interventions, and after discharge from the ICU, the patient reported significant difficulties in mobility, quality of life, and mental health.

This case prompted a focus on the prevention of ECOM-related foot drop complications and the importance of continuity of care for patients with PICS based on patient user profiling, journey mapping. In order to prevent similar events, ensure patient safety and improve quality of life.

## 2. Case presentation

Patient, 17 years old female, sophomore in high school, an athlete, with a weight of 53 kg, a height of 167 cm, and a BMI of 19, had a history of hypertension for 2 months. The family is a reconstituted family, a nuclear family, with a half-sister from the same father and a full sister from both parents. The family is harmonious. The patient has no other previous health problems, no psychosocial problems, and no genetic disorders.

**Figure 1.**
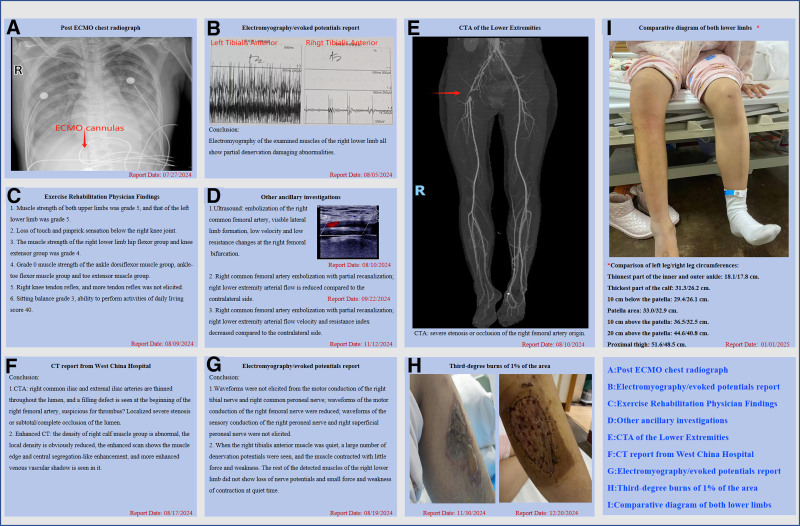
Clinically relevant findings. A comprehensive summary of the patient’s clinical findings following ECMO, detailing the severity of lower extremity drop foot complications and rehabilitation assessments. Report dates range from July 25, 2024 to January 1, 2025, indicating the longitudinal tracking of the patient’s condition. ECMO = extracorporeal membrane oxygenation.

After taking a large amount of antihypertensive medication for high self-measured blood pressure, she was transferred to the ICU after gastric lavage treatment in the Emergency Department on July 25, 2024, and was admitted with a diagnosis of heart failure, which required V-A ECMO treatment. She was treated in the ICU for a total of 15 days, including 3 days on ECMO and 6 days of invasive ventilator-assisted ventilation. After the withdrawal of the machine, the patient gradually regained voluntary consciousness and reported burning and pinprick pain in the right lower limb with sensory-motor deficits. After 2 days of high-flow nasal cannula oxygen therapy, intermittent nasal plug oxygen therapy was given. She was then transferred to the rehabilitation department for 9 days and then to a private rehabilitation hospital for 30 days. She was then treated for fever in the general ward for 54 days and returned home. A third-degree burn with an area of 1% occurred at home while using an electric stove for heating, and she was referred to the burns and plastic surgery department for closed negative pressure drainage and skin grafting. She was discharged after 22 days and returned to the community to continue her rehabilitation treatment. Clinically relevant findings during hospitalization are shown in Figure 1. The diagnosis of lower extremity drop foot was confirmed by clinical electromyography, third-degree burns were assessed using the burn assessment criteria, and the scale confirmed PICS.

**Figure 2.**
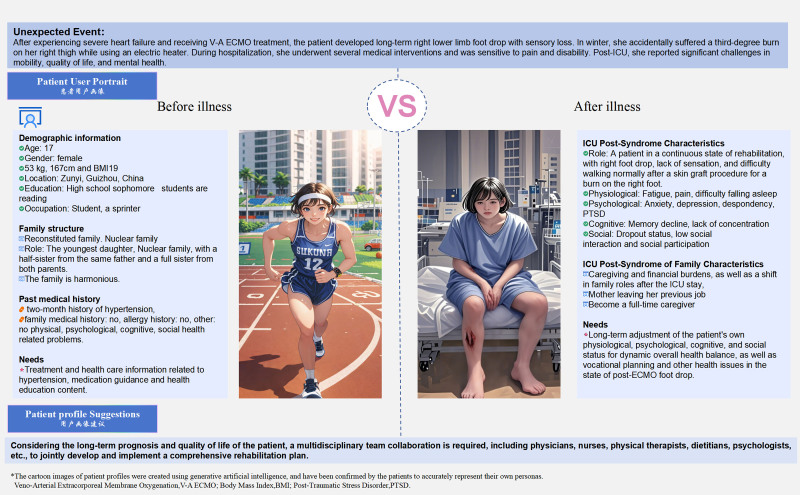
Patient user portrait before and after illness. Illustrates the patient’s detailed demographic and health-related profile changes in physiological, psychological, cognitive, and social aspects before and after the illness.

**Figure 3.**
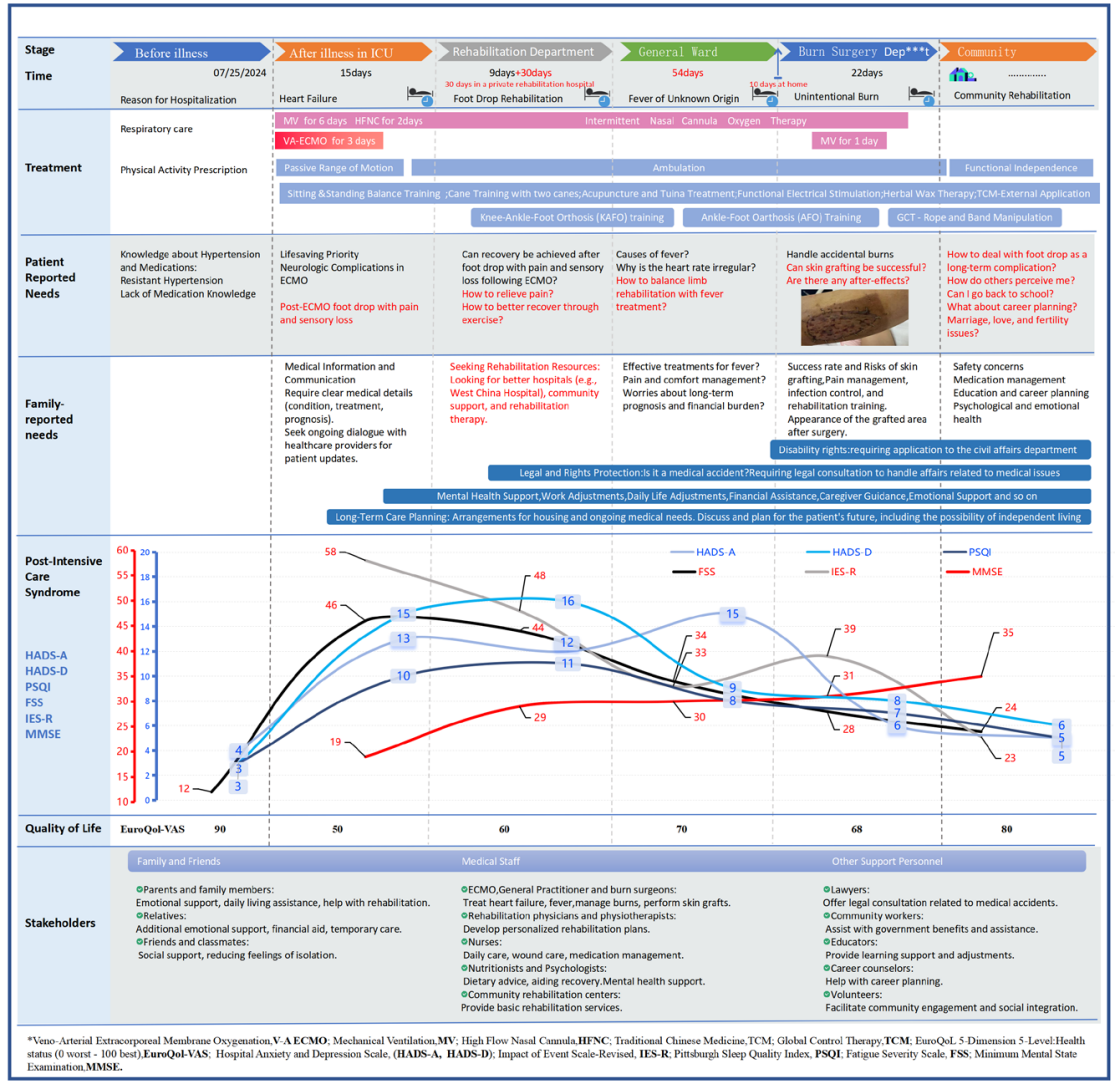
Patient health management journey map. A detailed chronological description of the patient’s health management process from ICU admission to community reintegration is presented, including the key treatment interventions at each stage, the needs of the patient and family, and the relevant stakeholders and roles involved. The chart also integrates patient PICS symptoms (physical symptoms, mental status, and cognitive functioning) and health-related quality of life (health status) over time. This mapping serves as a visual tool to help understand the complex care pathways for patients with complications after ECMO, emphasizing the interplay of medical, psychological, and social factors that influence patient recovery and overall well-being. ECMO = extracorporeal membrane oxygenation, PICS = post-intensive care syndrome.

The patient experienced several medical interventions and had significant PICS symptoms after discharge from the ICU, we used ongoing field interviews, which is a qualitative research method. The needs of patients and their families at different stages were found. In addition, it was found that patients reported significant health problems in terms of anxiety, depression, fatigue, cognition, post-traumatic stress disorder, sleep, and quality of life through a quantitative research method-continuous questionnaire follow-up. PICS-related scales and quality of life scale are shown in Annex 1, Supplemental Digital Content, https://links.lww.com/MD/P715. A user portrait of the patient before and after the illness is drawn as shown in Figure 2. The patient health management journey map is shown in Figure 3.

## 3. Follow-up and outcomes

Despite comprehensive intervention, the patient showed only partial recovery of foot function. Long-term permanent non-rehabilitable nerve injury complications of foot drop persisted and accidental burns occurred. However, as shown in the findings for each stage of PICS in Figure 3: journey map, all of the PICS-related symptoms improved significantly after returning to the community compared to the period of stay in the ICU, but there was still a difference compared to the pre-sickness period, with an improvement in overall physical health from a score of 50 during the ICU period to a score of 80.

The patient was provided with comprehensive coverage of therapeutic interventions by a multidisciplinary team during hospitalization and discharge rehabilitation. This included a variety of aspects such as emergency treatment, life support, rehabilitation training, psychological support, nutritional management, pain management, daily care, community rehabilitation, legal counseling, and educational and vocational planning.

Specifically, it includes early implementation of life-support measures such as V-A ECMO and mechanical ventilation, setting immediate and long-term rehabilitation goals for foot drop, such as: pain relief of <3 points within 2 weeks, activities of daily living score of 55, pain reduction of <2 points within 1 month, right lower limb muscle strength of 5, right knee flexion/extension range of 5° to 90°, and activities of daily living score of 80. And physical therapy such as passive joint mobility and walking training, including but not limited to not limited to sitting & standing balance training; cane training with 2 canes; acupuncture and tuina treatment; functional electrical stimulation; herbal wax therapy; traditional Chinese medicine (TCM) external application, knee-ankle-foot orthosis (KAFO) training, ankle-foot oarthosis (AFO) training, global control therapy—rope and band manipulation technique. Medications such as nerve growth factors and vitamins were also administered to promote neurological recovery. Burns were treated with skin grafts, and the patient also received peri-rehabilitation mental health and nutritional support, management of wounds and medications, as well as assistance with community rehabilitation and legal advocacy. Family and friends provided emotional support, practical help, social interaction and counseling during the patient’s recovery, such as encouragement from classmates’ Maple Leaf cards during the period and visits to reduce loneliness. The nurse-led writing of an electronic ICU diary improves the patient experience, etc. It also involves educators and career counselors to help patients plan their studies and career development, and volunteers to promote their community participation and social integration, which together build a multi-dimensional holistic care system aimed at comprehensively improving patients’ treatment outcomes and quality of life.

## 4. Discussion

### 4.1. Causes of foot drop and countermeasures

Possible mechanisms of foot drop due to unilateral neurologic impairment include nerve injury due to puncture, obstruction of femoral artery flow due to arterial cannulation, patient-specific factors (e.g., age, underlying disease, and vascular status), and treatment-related factors (e.g., inadequate anticoagulant therapy, slowed blood flow, and the use of vasoactive medications, and poor blood pressure control). In addition, femoral artery cannulation may lead to lower extremity ischemia from arterial entrapment or localized plaque dislodgement, resulting in distal arterial embolism or distal perfusion blockage.^[6–9]^ Typical symptoms include pain, abnormal sensation, paresthesia, pale or cyanotic skin, decreased skin temperature, and diminished or absent dorsalis pedis arterial pulsation.^[10]^ Routine assessment of the risk of lower limb ischemia, such as capillary filling test, transcutaneous partial pressure of oxygen, color Doppler ultrasonography, and pulse oximetry, selection of the appropriate type and size of cannulae, intensification of anticoagulation, and careful observation of the patient’s lower limbs are all recommended for the timely detection and management of ischemic signs.^[6]^ The use of distal perfusion catheters can effectively reduce the incidence of limb ischemia.^[11,12]^

### 4.2. Incidence of ECMO-related foot drop and rehabilitation programs

Bergeron retrospective study showed a 7.8% (12/153) incidence of foot drop in patients with V-A ECMO who received femoral artery cannulation support for more than 1 hour, of whom (5/12) were unable to walk.^[13]^ This suggests that foot drop not only affects quality of life but also requires long-term physiotherapy and rehabilitation interventions. Foot drop is still considered as one of the rare neurological complications associated with V-A ECMO.^[14]^ Rehabilitation needs to be comprehensive and gradual, and rehabilitation options include medication: for example, neurotrophic, anti-inflammatory and analgesic medications; physical therapy: functional electrical stimulation, herbal warm compresses, and Chinese acupuncture; surgical treatment: nerve release, suturing; and other adjunctive integrative therapies: ankle-foot orthosis, global control therapy—rope and band manipulation technique, which helps to maintain a normal foot position and helps to walk more safely through external forces.

### 4.3. Prevention of foot drop with sensory and motor impairment accidents

Abnormal gait due to dysfunction makes it easy to lose balance while walking, increasing the risk of falls and falling out of bed.^[15]^ Loss of perception and sensation, poor limb placement with pressure injuries and other skin problems, especially insensitivity to temperature stimuli increases the risk of burns, thus the need for health education on the prevention of secondary accidents.

### 4.4. The role of user profiling and patient health journey mapping’s in PICS after ECMO therapy

After undergoing ICU hospitalization for ECMO treatment, patients may experience PICS, which may cause them to experience new problems or exacerbation of existing problems in physical, cognitive, and psychological areas, and these effects may persist after discharge, affecting patients and their families.^[5,16]^ To effectively manage PICS, healthcare teams utilize 2 modern healthcare management tools: patient user profiles and health journey maps. User profiles enable the healthcare team to identify the patient’s core needs and potential risk factors so that a personalized recovery plan can be developed.^[17,18]^ The health journey map, on the other hand, depicts the patient’s entire recovery process from admission to discharge, identifying key interventions and expected outcomes at each stage to ensure that the patient receives appropriate care and support throughout the recovery process.^[19,20]^ The combined use of these 2 tools can help reduce the incidence of PICS and enhance the quality of a patient’s recovery, as well as ensure that relevant stakeholders have a deeper understanding of the patient’s individual needs and provide them with a more accurate holistic program.

## 5. Conclusion

Unilateral neurological complications such as foot drop with sensorimotor deficits after ECMO are rare but can significantly impact patient quality of life. In this case, the subsequent burn injury resulted from the functional impairment caused by foot drop; although not a direct complication of ECMO, it further aggravated the patient’s physical condition and underscores the importance of attentive long-term rehabilitation care. This case report combined with patient user profiles and journey maps provides a new vision management framework for healthcare providers to better understand and manage post-ECMO neuromuscular complications and PICS. It highlights the importance of a comprehensive patient-centered integrated program with multidisciplinary collaboration.

## Acknowledgments

Special thanks go to the patient and their family for authorizing the report.

## Author contributions

**Conceptualization:** You Yuan, Jingwen Zhang.

**Data curation:** You Yuan, Jingwen Zhang, Yanyan Mou, Li Li, De Su, Feiyu Yan.

**Formal analysis:** You Yuan, Yan Zhou, Chaojin Yang, De Su, Feiyu Yan.

**Funding acquisition:** Yan Zhou, Chaojin Yang, Rujun Hu.

**Investigation:** You Yuan, Yanyan Mou, Chaojin Yang, Li Li.

**Methodology:** Yanyan Mou, Chaojin Yang.

**Project administration:** Yan Zhou, Chaojin Yang, Huiming Gao, De Su, Rujun Hu.

**Visualization:** Huiming Gao.

**Writing – original draft:** You Yuan, Jingwen Zhang.

**Writing – review & editing:** Rujun Hu.

## Supplementary Material


