# Supplementary material for: Drop foot post-ECMO, subsequently complicated by third-degree burns: A case report based on user portrait and health management journey map
Source: Medicine (Baltimore). 2025 Aug 22;104(34):e44008. doi: 10.1097/MD.0000000000044008 (PMC12384982; doi:10.1097/MD.0000000000044008)
Supplement: Supplementary file 1 [file medi-104-e44008-s001.docx]

**Post-ICU Syndrome(PICS)-related Survey Scales and Quality of Life Assessment Scale**

**HADS-A:** Hospital Anxiety and Depression Scale - Anxiety is a subscale of the HADS scale used to assess anxiety status, containing 7 entries, each entry is rated on a 4-point scale according to the frequency of symptom occurrence in the last month, with a total scale score of 0 to 28 points, with higher scores indicating more severe anxiety symptoms. Abnormal scores were ≥8.

**HADS-D:** Hospital Anxiety and Depression Scale - Depression is a subscale of the HADS scale used to assess depressive states, also containing 7 entries and scored on the same scale as the HADS-A. The HADS-D is a subscale of the Hospital Anxiety and Depression Scale (HADS) used to assess depressive states.

**PSQI:** Pittsburgh Sleep Quality Index is used to assess sleep quality. The scale contains several components such as sleep duration, sleep efficiency, and sleep disorders. The scale is scored on a scale of 0 to 21. The higher the score the poorer the quality of sleep. Abnormal scores are ≥7.

**FSS:** Fatigue Severity Scale is a scale used to assess the severity of fatigue and consists of 9 entries on a 7-point scale ranging from 1, “not at all severe” to 7, “very severe”. The total scale score ranges from 9 to 63, with higher scores indicating more severe fatigue. Abnormal scores were ≥36.

**IES-R:** Impact of Event Scale-Revised is a 22-item scale divided into three subscales: intrusion, avoidance, and excessive arousal. Scale entries are rated on a 5-point scale from 0-4. The total scale score is 0-88, with higher scores indicating greater impact of the event. Abnormal scores were ≥35.

**MMSE:** Mini-Mental State Examination is a scale used to assess cognitive dysfunction, containing 7 aspects of time orientation, place orientation, immediate memory, attention and calculation, delayed memory, language, and visuospatial, with a total of 30 items, each correct answer scores 1 point, and incorrect answers or don't know rate 0 points, with a total scale score ranging from 0-30 points. Abnormal scores were ≤25 points.

**EQ-VAS:** EuroQol-Visual Analogue Scale, the EuroQol-5D-5L contains a visual analogue scale (EQ-VAS) for respondents to self-assess their current general state of health, ranging from 0 (worst state of health) to 100 (best state of health).
